# Supplementary material for: Autotaxin–lysophosphatidic acid–LPA 3 signaling at the embryo‐epithelial boundary controls decidualization pathways
Source: EMBO J. 2017 Jun 6;36(14):2146–60. doi: 10.15252/embj.201696290 (PMC5509998; doi:10.15252/embj.201696290)
Supplement: Supplementary file 1 — Appendix [file EMBJ-36-2146-s001.pdf]

Appendix

Appendix figures

Appendix Figure S1. Alkaline phosphatase (AP) activity in T13-injected uteri.
Appendix Figure S2. Pharmacological inhibition of EGFR, COX-2 and ERα don't decrease the T13-induced expression of *Hbegf* and *Ptgs2*.
Appendix Figure S3. LPA detection in the uterine flushing fluids from the pregnant mice.
Appendix Figure S4. Detection of LPC species in pre-implanted blastocysts.
Appendix Figure S5. Activation of LPA_3 induced ectodomain-shedding of HB-EGF *in vitro*.
Appendix Figure S6. Uterine tissue from *Lpar3* KO mice less developed in Endometriosis model.

Appendix Methods
Appendix References

Figure S1

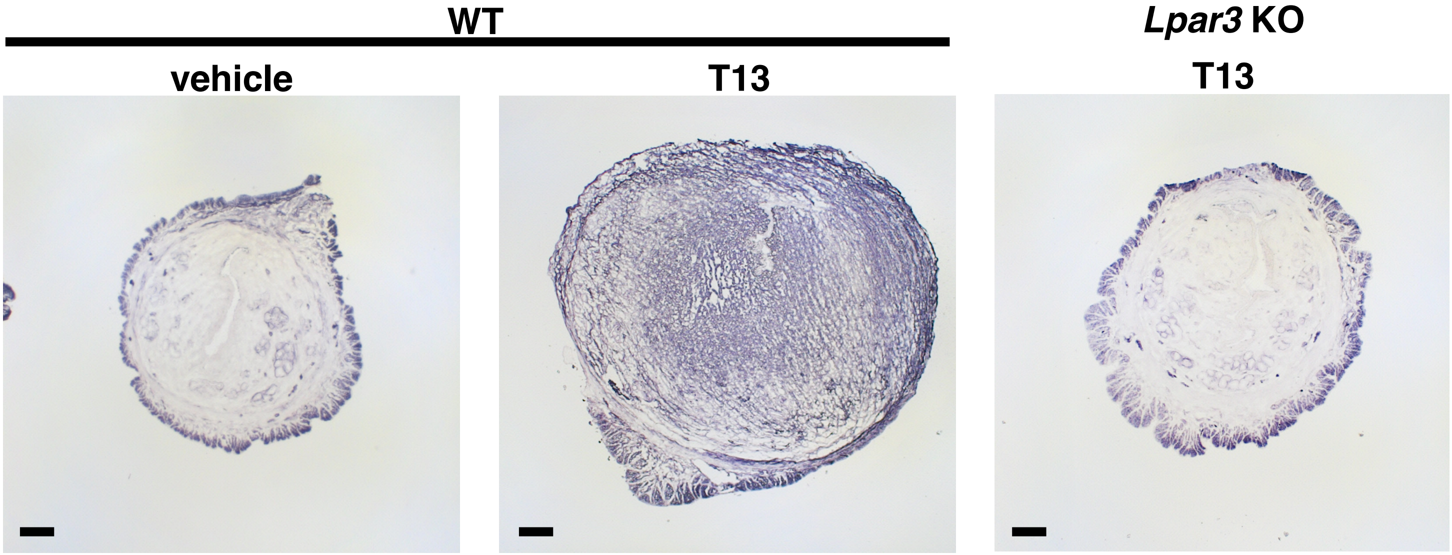

Figure S1. Alkaline phosphatase (AP) activity in T13-injected uteri.
Representative photographs of AP staining in T13-injected uteri 48 h after the injection. High AP activity was induced by T13-injection in an LPA_3-dependent manner. Each image is a representative from at least 3 independent experiments. Scale bar = 200 μm.

Figure S2

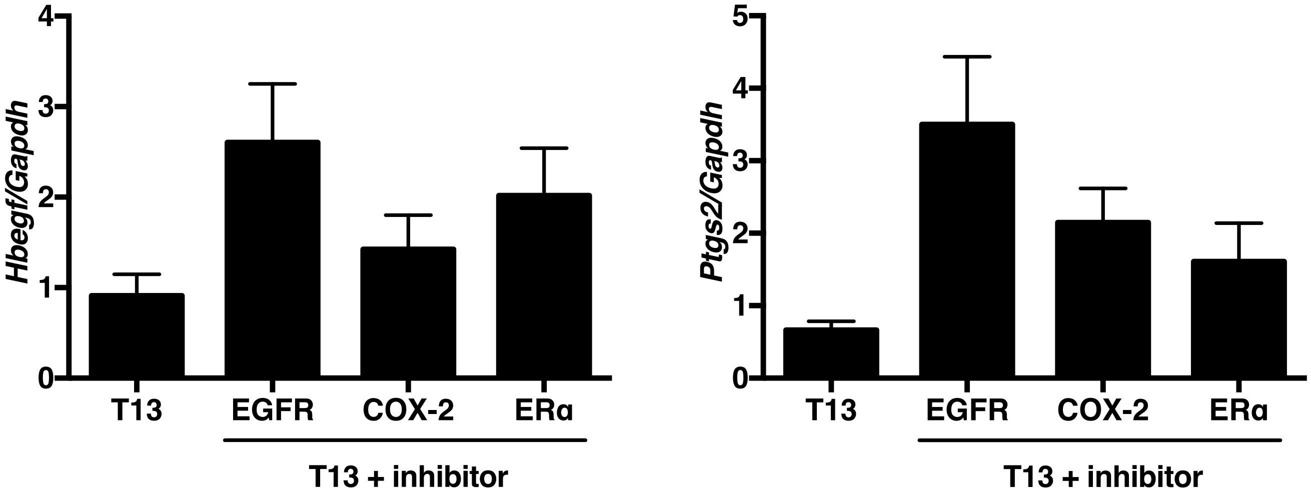

Figure S2. Pharmacological inhibition of EGFR, COX-2 and ERa don't decrease the T13-induced expression of *Hbegf* and *Ptgs2*.

Six hours after the treatment of T13 and inhibitors, each expression of *Hbegf* and *Ptgs2* was evaluated by qRT-PCR (n = 20 for T13,n = 9 for T13 + EGFR inhibitor, n = 10 for T13 + COX-2 inhibitor and n = 13 for T13 + ERα antagonist in *Hbegf*; n = 9 for T13, T13 + EGFR inhibitor and T13 + ERa antagonist, n =10 for T13 + COX-2 inhibitor in *Ptgs2*). Data are means + SEM.

Figure S3

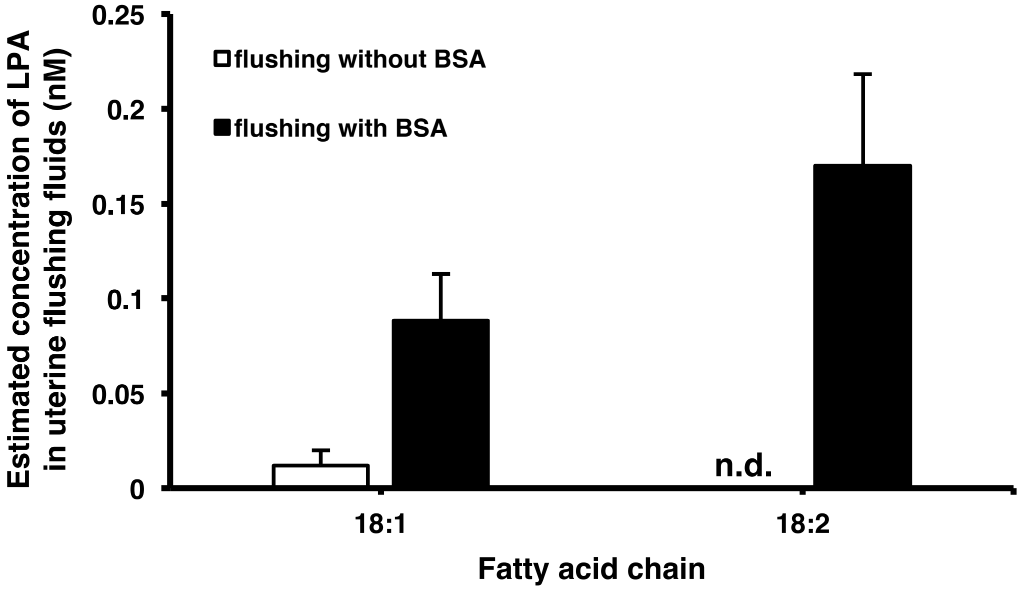

Figure S3. LPA detection in the uterine flushing fluids from the pregnant mice.
The pregnant uteri were flushed with 50 μl of saline with or without albumin. 30 μl of the flushed fluids were mixed with 70 μl methanol and then processed for LC-MS/MS analysis (n = 5 for each bar). When the uteri were flushed with the albumin-containing saline, LPA with an unsatulated fatty acid was detected. The estimated concentration of LPA in the uterine cavity is 1-2 nM, provided that the volume of uterine cavity is ~5 μl. Data are means + SEM, n.d.: not detected.

Figure S4

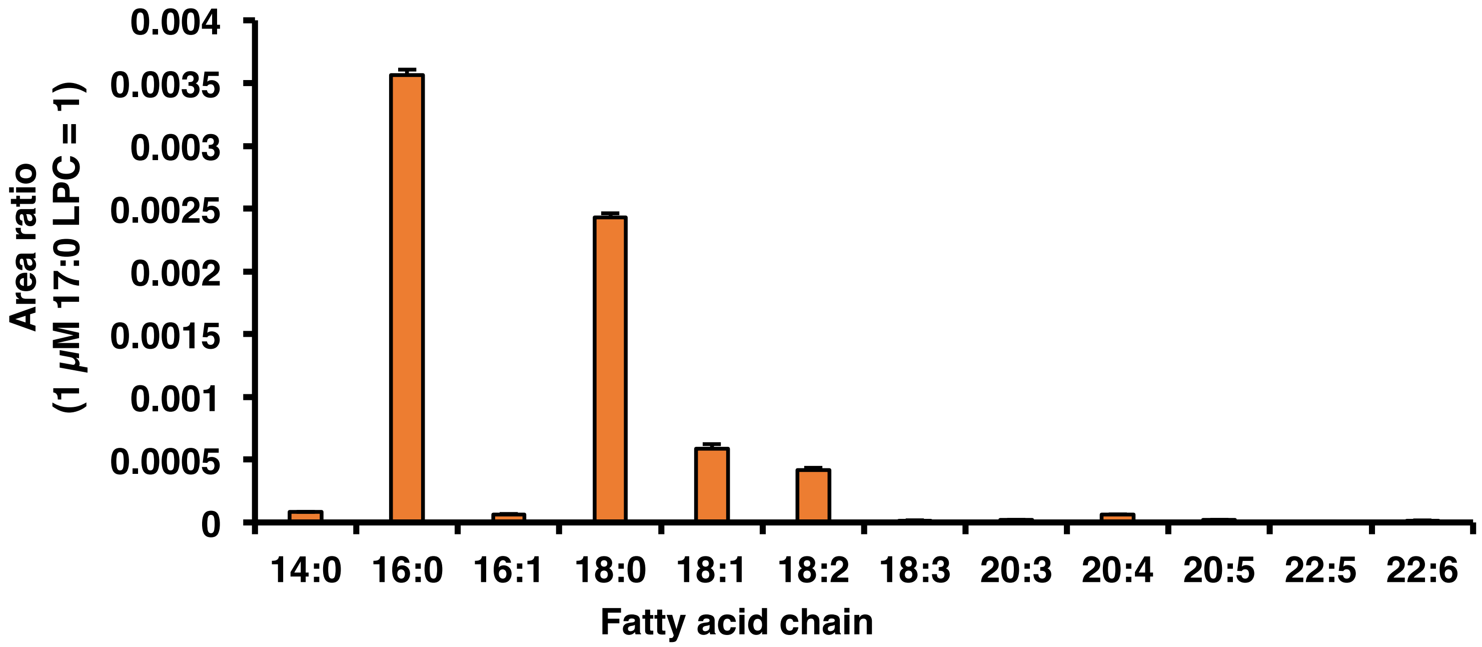

Supplementary Figure S4. Detection of LPC species in pre-implanted blastocysts.
Ten pre-implanted embryos were mixed and subjected to lysophospholipid analysis using LC-MS/MS. A single data point was measured by two biological replicates. Representative data from two experiments with similar results were shown. Data are means + SD.

Figure S5

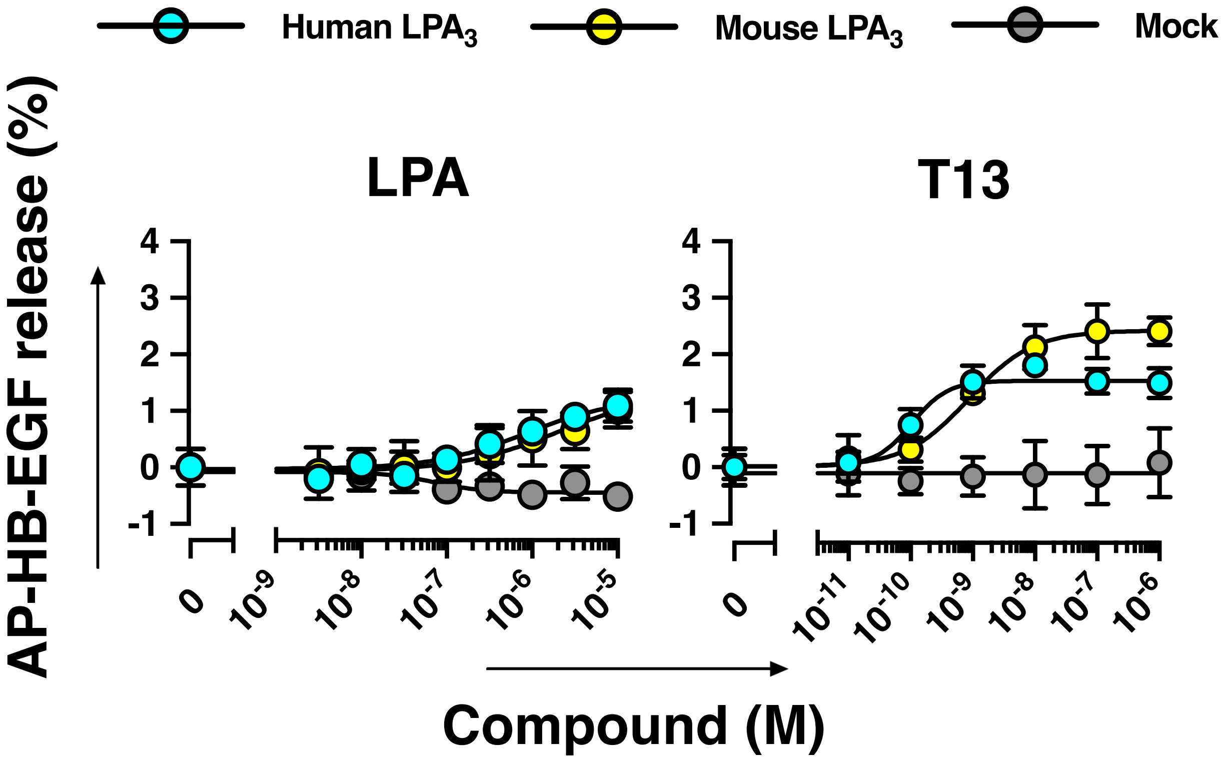

Figure S5. Activation of LPA_3 induced ectodomain-shedding of HB-EGF *in vitro*.
LPA_3 and AP-HB-EGF plasmids were co-transfected to HEK293 cells which endogeneously express some protease responsible for ectodomain-shedding of HB-EGF. LPA_3 was activated by LPA or T13 and then the shedding was evaluated by a method based on TGFa shedding assay (Inoue *et al*, 2012; see also “Materials and Methods” in the main manuscript). For each experiment, a single data point was measured by three biological replicates. Data are means ± SEM, respectively.

Figure S6

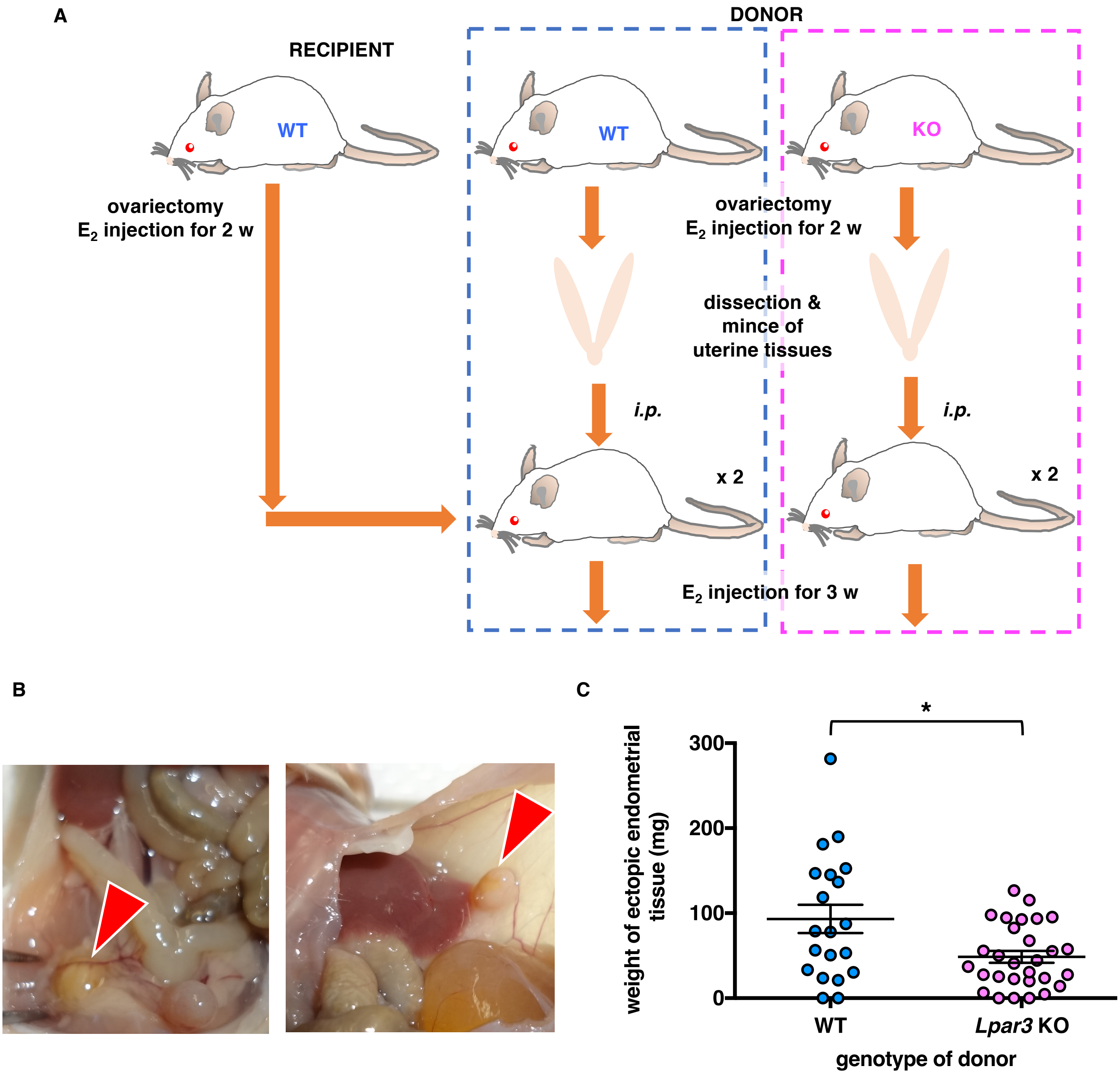

Figure S6. Uterine tissue from *Lpar3* KO mice less developed in Endometriosis model.
(A) Scheme of the endometriosis model. The uterine tissues from donor mice (either WT or *Lpar3* KO) were minced and injected *i.p.* into WT recipient mice (n = 28 for WT and n = 30 for *Lpar3* KO), and three weeks after, weight of endometrial tissues developed in the peritoneal cavity was determined. Eight recipient mice for WT uterine tissues and one for *Lpar3* KO uterine tissues died during the three weeks. (B) Representative photographs of ectopic endometrial tissues developed in peritoneal cavity (arrow heads). (C) The weight of ectopic endometrial tissues in survived mice three weeks after the injection of endometrium. Data are means ± SEM, $^{*}P < 0.05$.

Appendix Methods

Alkaline phosphatase staining

Alkaline phosphatase staining was performed as previously described (Franco *et al*, 2011) with some modifications. Briefly, 8 µm cryosections were fixed in 0.2% glutaraldehyde and then washed in PBS. Sections were incubated with a 100 mM Tris buffer (pH 9.5) containing 5-bromo-4-chloro-3-indolyl phosphate and nitro blue tetrazolium chloride (Roche Applied Science).

LC-MS/MS analysis of LPA and LPC

The pregnant uteri on 3.5 dpc were flushed with 50 µl of saline with or without 0.1 % bovine serum albumin (fatty acid-free, Sigma-Aldrich) through the tubal end. The flushing fluids (30 µl) were mixed with 70 µl methanol with 17:0 LPA as an internal standard. The blastocysts were recovered at 3.5 dpc from the uteri by flushing with saline through the tubal end. The recovered embryos were then collected using a glass micropipette (Drummond Scientific Company) under a dissecting microscope. After centrifugation (190 g for 5 min), the blastocysts pellets were mixed with methanol (100 µl) with 17:0 LPC as an internal standard.

Each sample was homogenized for 10 min in an ultrasonic bath and centrifuged at 21,500 g for 10 min at 4 °C. The supernatant was passed through a filter (0.2 µm pore size, 4 mm inner diameter; YMC). Samples were injected into a Dionex UltiMate 3000 (Thermo Fisher Scientific) with the columns of Capcell Pak C18 (150 mm × 0.3 mm i.d., 3 µm particle size) as analytical column and Capcell Pak C18 (50 mm × 0.3 mm i.d., 3 µm particle size) as trap column. Each column was maintained at 40 °C and the mobile phase flow rate was 10 µl/min. The mobile phase was same as detailed in an earlier study (Okudaira *et al*, 2014). The MS system was a TSQ Quantiva (Thermo Fisher Scientific) triple quadrupole mass spectrometer equipped with a heated-electrospray ionization-II (HESI-II) source. HESI was performed in the positive mode for LPC and negative mode for LPA. Samples were analyzed in SRM mode, using the transitions of the [M+H]+ precursor ions to their product ions. The concentration of LPA in the uterine flushing fluids was calculated by comparing the peak area of each LPA species with the area of internal standard (17:0 LPA, final 70 nM in each sample).

Endometriosis model

Mouse endometriosis model was performed according to the method previously reported (Hirata *et al*, 2005; Tomio *et al*, 2013). First, both donor (*Lpar3* KO or WT mice, 6–8 weeks old) and recipient mice (WT mice, 6–8 weeks old) were subjected to ovariectomy to eliminate the effects of endogenous female sex hormones. After the ovariectomy, all donor mice and recipient mice were injected s.c. with b-estradiol (100 mg/kg, Wako Pure Chemical Industries) in corn oil every week. Two weeks after the ovariectomy, uterine horns were dissected from the donor mice and minced using a scissor and a razor blade. Uterine tissue fragments suspended in PBS were injected with an 18-gauge needle into the peritoneal cavity of the recipient mice with a ratio of one donor to two recipients. Three weeks after the injection, the recipient mice were sacrificed and peritoneal endometriotic lesions were measured directly by weighing.

Appendix References

Franco HL, Dai D, Lee KY, Rubel CA, Roop D, Boerboom D, Jeong JW, Lydon JP, Bagchi IC, Bagchi MK, DeMayo FJ (2011) WNT4 is a key regulator of normal postnatal uterine development and progesterone signaling during embryo implantation and decidualization in the mouse. FASEB J 25: 1176-87

Hirata T, Osuga Y, Yoshino O, Hirota Y, Harada M, Takemura Y, Morimoto C, Koga K, Yano T, Tsutsumi O, Taketani Y (2005) Development of an experimental model of endometriosis using mice that ubiquitously express green fluorescent protein. Hum Reprod 20: 2092-6

Inoue A, Ishiguro J, Kitamura H, Arima N, Okutani M, Shuto A, Higashiyama S, Ohwada T, Arai H, Makide K, Aoki J (2012) TGFα shedding assay: an accurate and versatile method for detecting GPCR activation. Nat Methods 9: 1021-9

Okudaira M, Inoue A, Shuto A, Nakanaga K, Kano K, Makide K, Saigusa D, Tomioka Y, Aoki J (2014) Separation and quantification of 2-acyl-1-lysophospholipids and 1-acyl-2-lysophospholipids in biological samples by LC-MS/MS. J Lipid Res 55: 2178-92

Tomio K, Kawana K, Taguchi A, Isobe Y, Iwamoto R, Yamashita A, Kojima S, Mori M, Nagamatsu T, Arimoto T, Oda K, Osuga Y, Taketani Y, Kang JX, Arai H, Arita M, Kozuma S, Fujii T (2013) Omega-3 polyunsaturated Fatty acids suppress the cystic lesion formation of peritoneal endometriosis in transgenic mouse models. PLoS One 8: e73085
