# Supplementary material for: Autotaxin–lysophosphatidic acid–LPA 3 signaling at the embryo‐epithelial boundary controls decidualization pathways
Source: EMBO J. 2017 Jun 6;36(14):2146–60. doi: 10.15252/embj.201696290 (PMC5509998; doi:10.15252/embj.201696290)
Supplement: Supplementary file 2 — Expanded View Figures PDF [file EMBJ-36-2146-s002.pdf]

Expanded View Figures

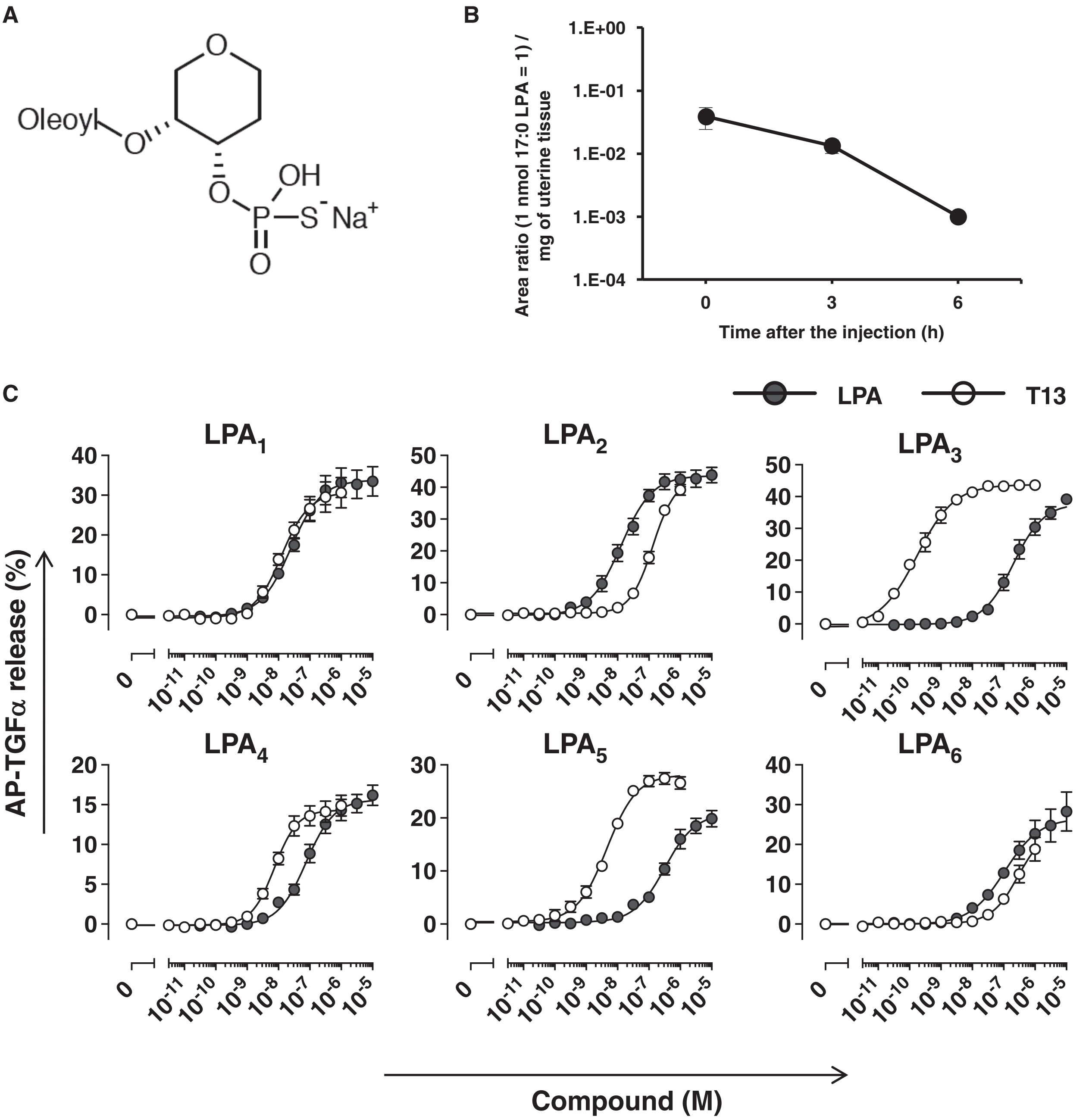

Figure EV1. T13 is a potent and selective agonist of LPA_3.

A The structure of T13. T13 was synthesized based on the structure of 2-oleoyl LPA and thiophosphate group and ring structure were introduced to make it more stable and resistant for phosphatase.

B The pharmacokinetics of T13 in uteri after the intrauterine injection. A single data point was measured in three biological replicates. Data are means ± SEM.

C Each LPA receptors and AP-TGFα plasmids were co-transfected to HEK293 cells which endogeneously express a protease, TACE, responsible for ectodomain-shedding of TGFα. Activation of each LPA receptor by T13 (open circles) and LPA (closed circles) was evaluated by TGFα shedding assay. T13 has a potent agonistic effect on LPA_3. For each experiment, a single data point was measured in three biological replicates. Receptor-specific responses were calculated by subtracting AP-TGFα release signals in mock-transfected cells from those in LPA receptor plasmid-transfected cells. Data are means ± SEM, respectively, of three (for LPA_1, LPA_2, LPA_3, and LPA_6) or four (for LPA_4 and LPA_5) independent experiments.

 © 2017 The Authors

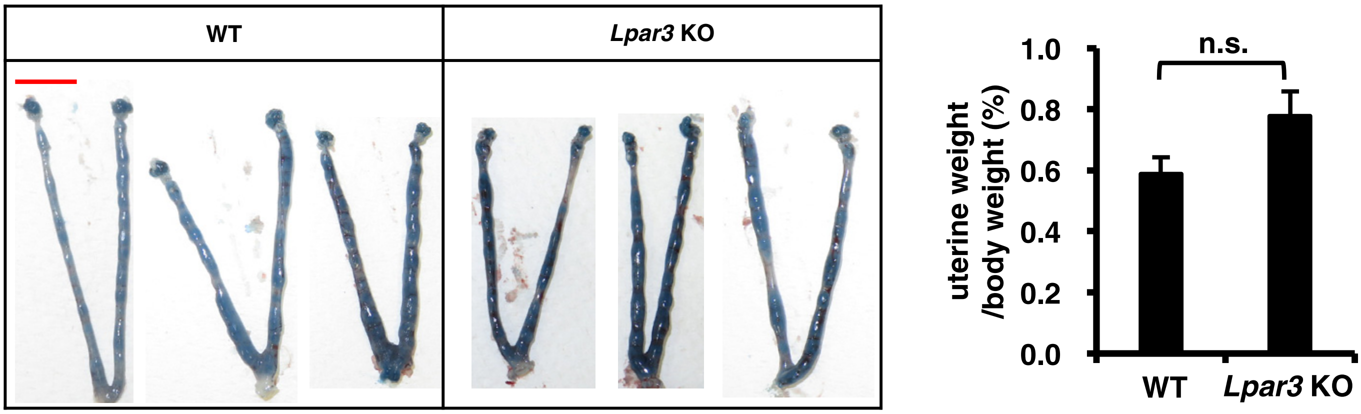

Figure EV2. Oil-induced decidualization is not affected in *Lpar3* KO mice.

Representative photographs (left) and the average mass of oil-infused uteri 2 days after the oil infusion (n = 10 for WT mice and n = 12 for *Lpar3* KO mice). Each image is a representative from at least three independent experiments. Scale bar: 1 cm. Data are means + SEM, n.s.: not significant by Student's t-test.

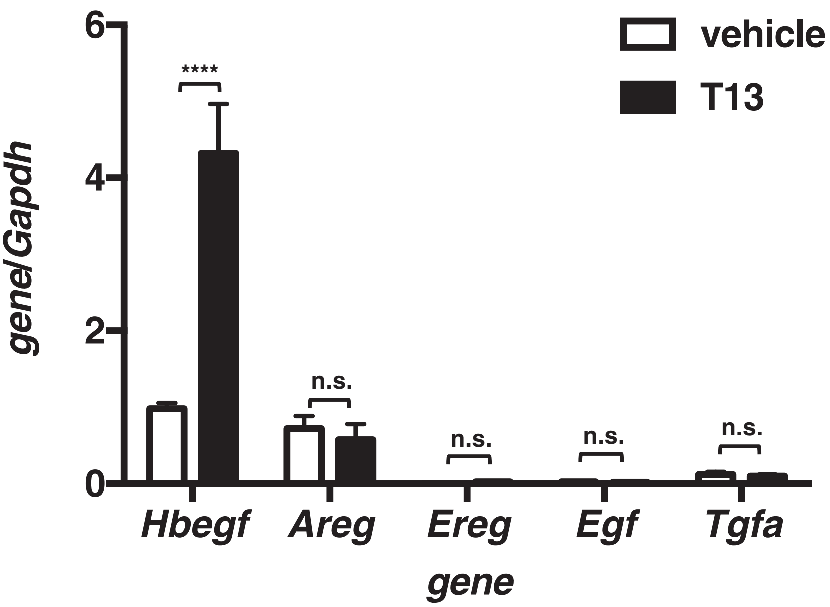

Figure EV3. T13 induces *Hbegf* specifically among the EGF family members.

Two hours after the T13 injection, expression of each EGF family member was evaluated by qRT–PCR (n = 5 for each bar). Data are means + SEM, ****$P < 0.0001$, n.s.: not significant by ANOVA.

© 2017 The Authors

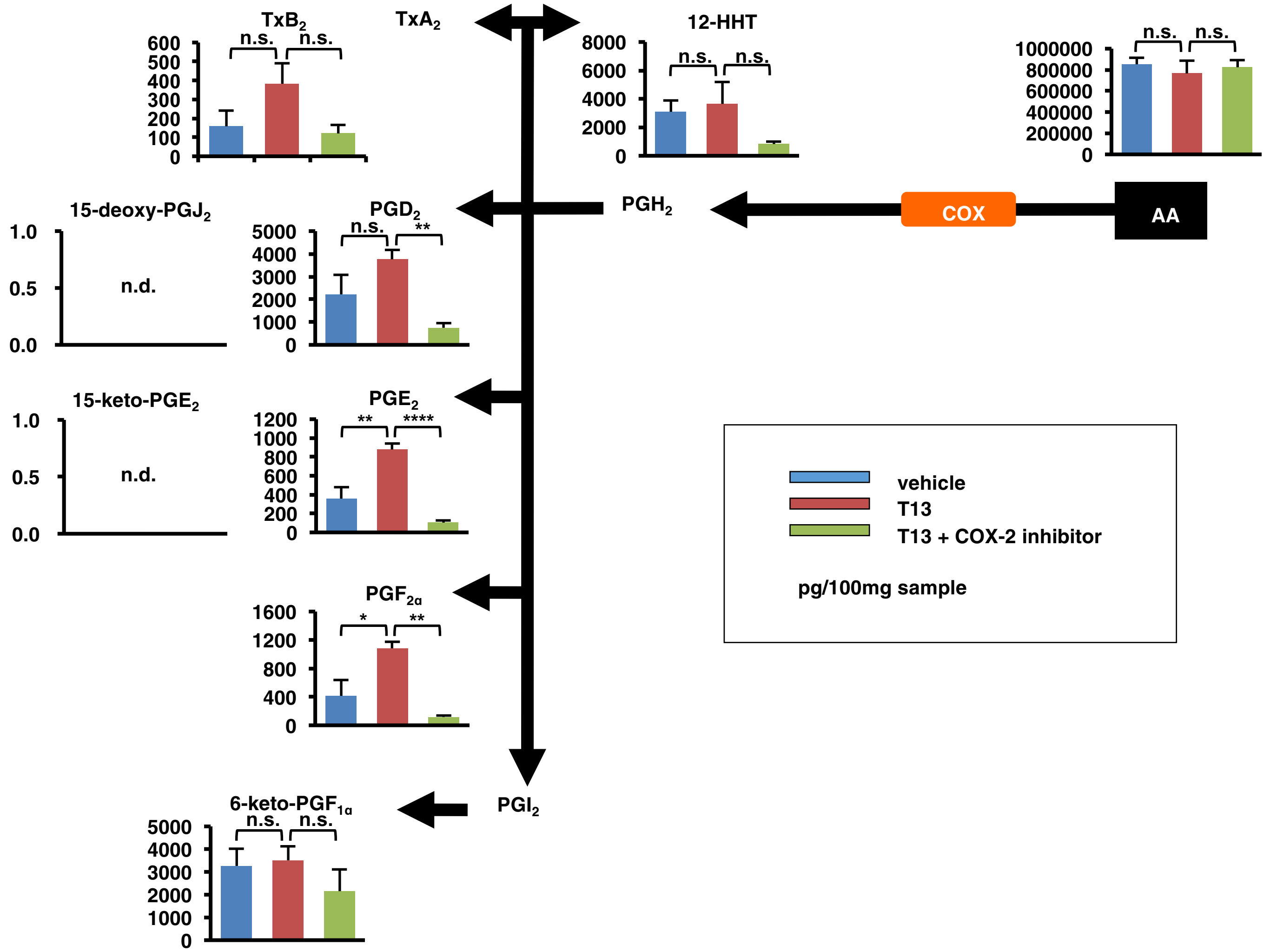

Figure EV4. Levels of prostaglandins and related lipids in T13-injected uteri.

Whole uteri were isolated from T13-injected mice 9 h after the T13 injection and were subjected to LC-MS/MS-based lipidomics analysis, mainly focused on fatty acids and their derivatives. Arachidonic acid (AA)-derived products as well as AA itself were quantified by LC-MS/MS. Effect of COX-2 inhibitor, Celecoxib, was also examined. In T13-injected uteri, PGE_2 and $PGF_{2\alpha}$ were significantly up-regulated in COX-2-dependent manner (n = 5 for each bar). Data are means + SEM. *$P < 0.05$, **$P < 0.01$, ****$P < 0.0001$, n.s.: not significant, n.d.: not detected. TxB_2: thromboxane B_2, TxA_2: thromboxane A_2, 12-HHT: 12-hydroxyheptadecatrienoic acid, 15-keto-PGE_2: 15-keto-prostaglandin E_2, 15-deoxy-PGJ_2: 15-deoxy-prostaglandin J_2, 6-keto-$PGF_{1\alpha}$: 6-keto-prostaglandin $F_{1\alpha}$.

© 2017 The Authors

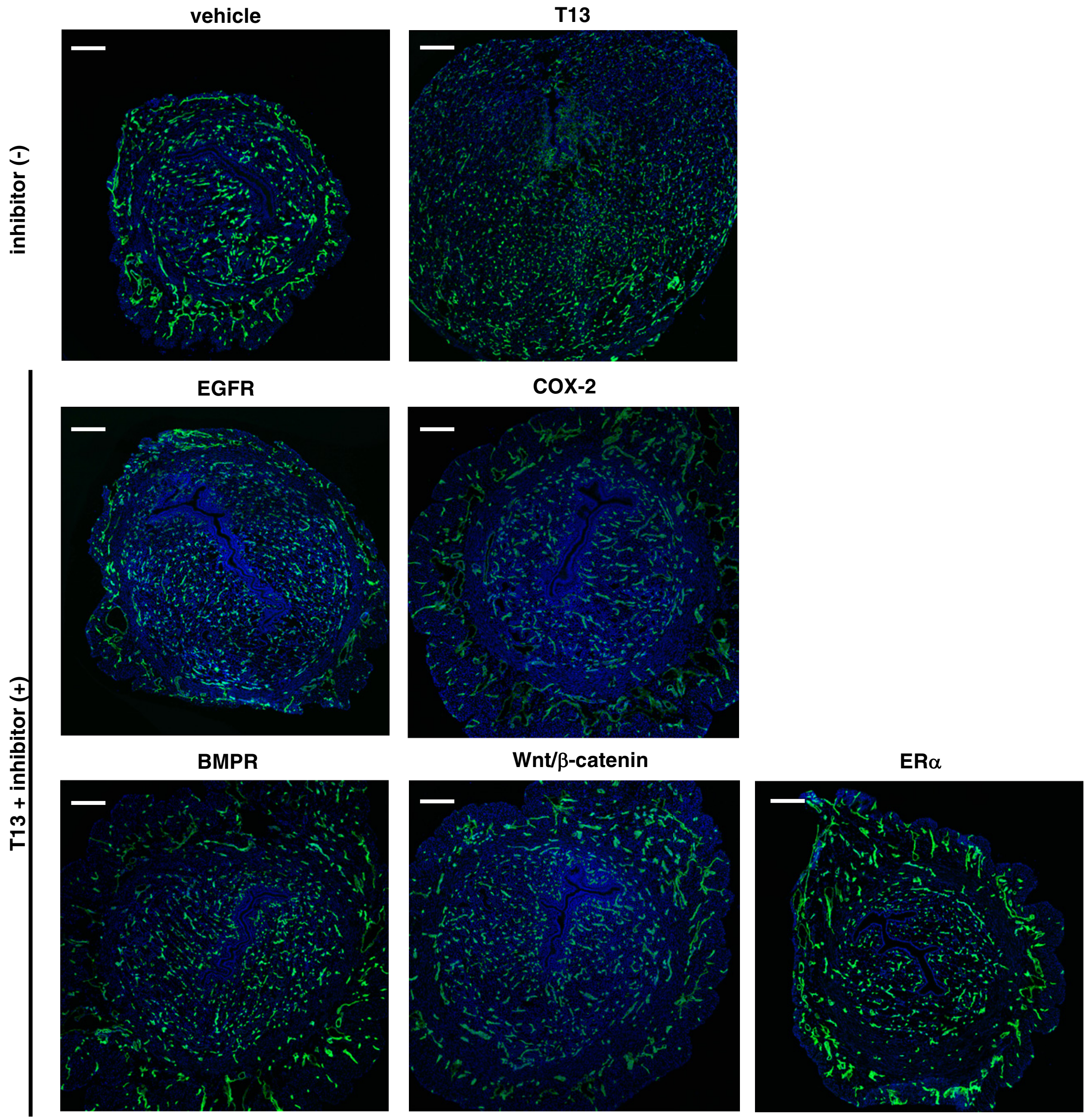

Figure EV5. Effects of each signal inhibitor on T13-induced uterine angiogenesis.

Immunohistochemical images of uterine cross-sections from pseudopregnant mice using anti-CD31 antibodies 2 days after the injection of both T13 and either one of the inhibitors. Each inhibitor prominently blocked T13-induced angiogenesis characterized by fine vascular formation in the AM pole. Scale bar: 200 μm.

© 2017 The Authors

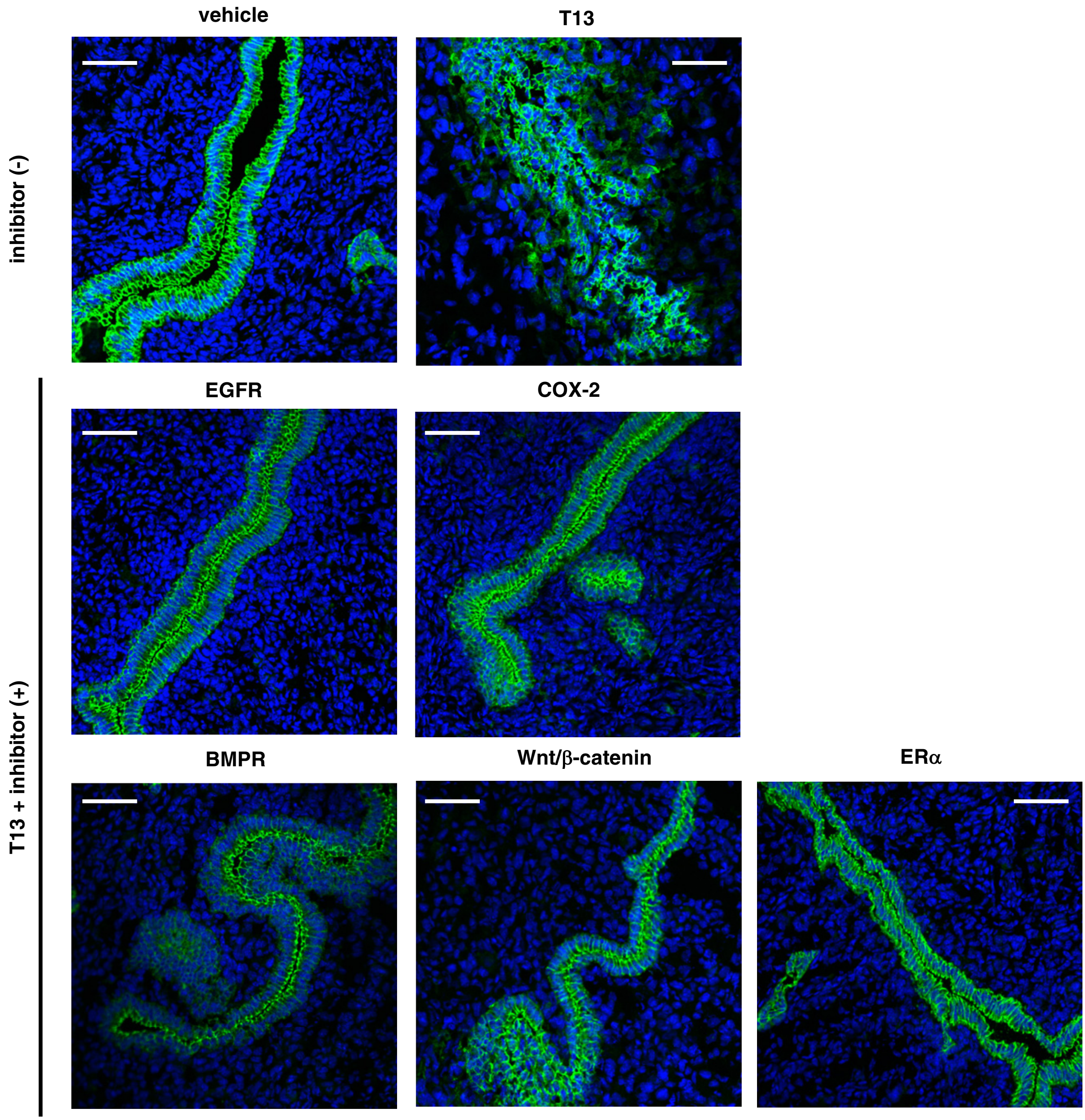

Figure EV6. Effects of each signal inhibitor on T13-induced LE-breakdown.

Immunohistochemical images of uterine cross-sections from pseudopregnant mice using anti-E-cadherin antibodies 2 days after the injection of both T13 and either of inhibitors, showing that T13-induced LE-breakdown was prominently blocked by each inhibitor. Scale bar = 50 μm.

 © 2017 The Authors

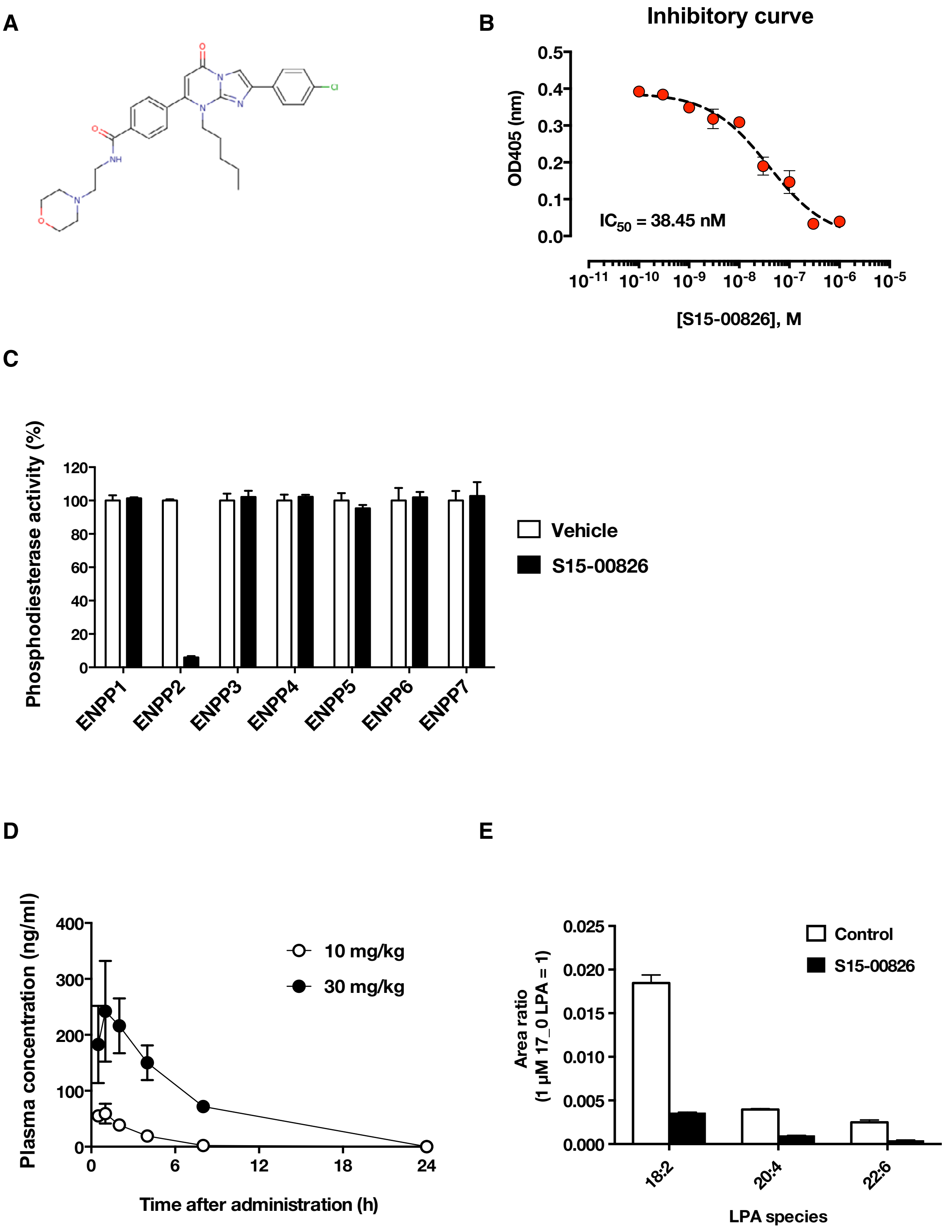

Figure EV7. The character of the ATX inhibitor.

A The chemical structure of S15-00826.

B Inhibitory curve of recombinant ATX by S15-00826. Data are means ± SD (n = 3). The IC_{50} value for S15-00826 is ~38 nM in ATX assay using p-nitrophenyl TMP as a substrate.

C The selectivity of S15-00826 was evaluated using phosphodiesterase activity assay. Among the six ENPP family members, S15-00826 inhibited the activity of ENPP2 (= ATX) selectively. A single data point was measured by two biological replicates. Data are means + SD.

D Pharmacokinetics in mice. The compound was *p.o.* administered to mice and collected bloods were analyzed in LC-MS/MS (n = 3 for each bar). Data are means ± SD.

E The effect of S15-00826 on circulating LPA in mice. The compound was administered to mice (20 mg/kg, *i.p.*), and plasma LPA level was determined by LC-MS/MS analysis. Data are means + SD (n = 3).

© 2017 The Authors
