# Supplementary figures and images for: Autotaxin–lysophosphatidic acid–LPA 3 signaling at the embryo‐epithelial boundary controls decidualization pathways
Source: EMBO J. 2017 Jun 6;36(14):2146–60. doi: 10.15252/embj.201696290 (PMC5509998; doi:10.15252/embj.201696290)

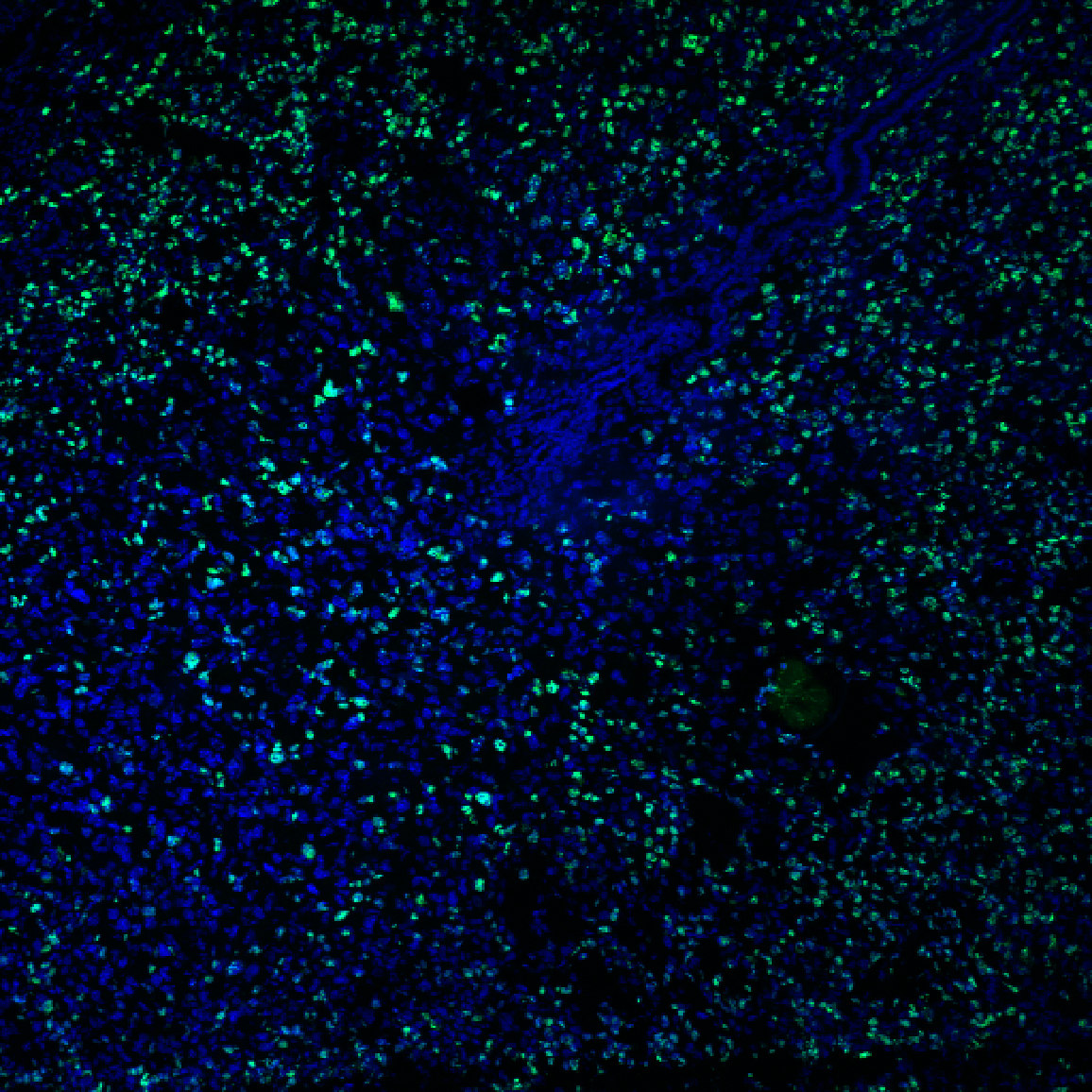

Supplement: Supplementary file 5 — Source Data for Figure 5 [file EMBJ-36-2146-s004.zip › Fig_5B_Source_Figures/5B_T13.tif]

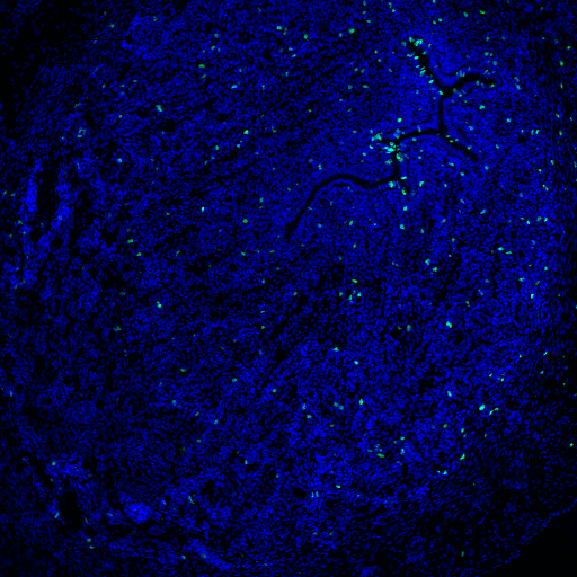

Supplement: Supplementary file 5 — Source Data for Figure 5 [file EMBJ-36-2146-s004.zip › Fig_5B_Source_Figures/5B_T13+BMPR.tif]

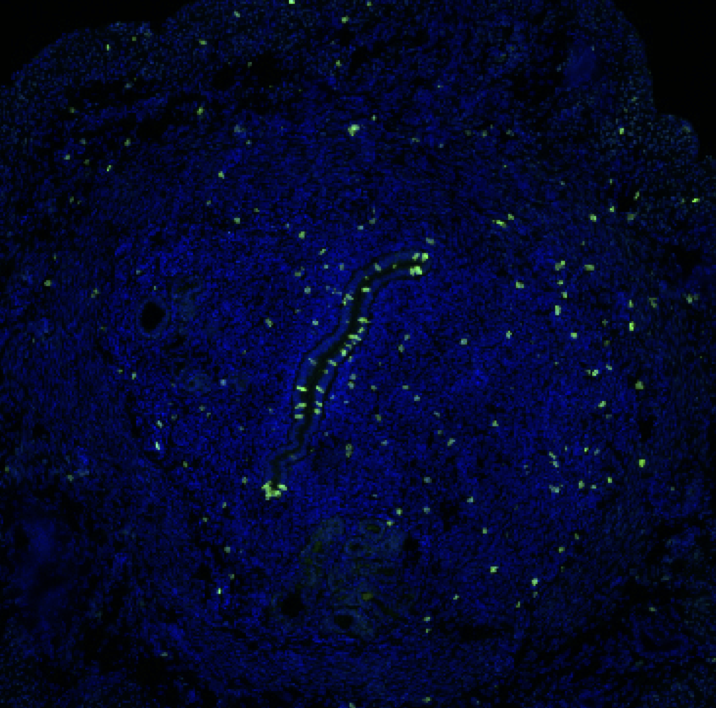

Supplement: Supplementary file 5 — Source Data for Figure 5 [file EMBJ-36-2146-s004.zip › Fig_5B_Source_Figures/5B_T13+COX-2.tiff]

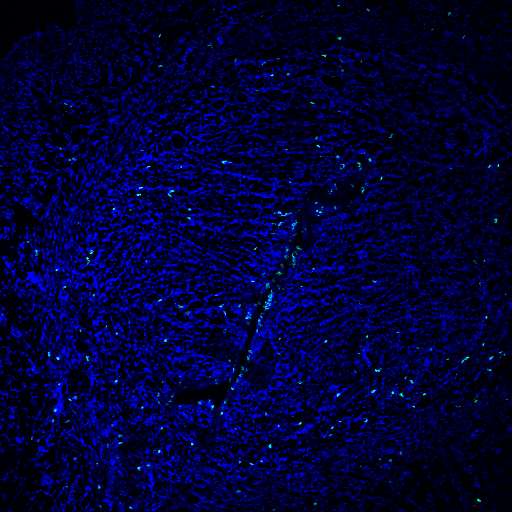

Supplement: Supplementary file 5 — Source Data for Figure 5 [file EMBJ-36-2146-s004.zip › Fig_5B_Source_Figures/5B_T13+EGFR.tif]

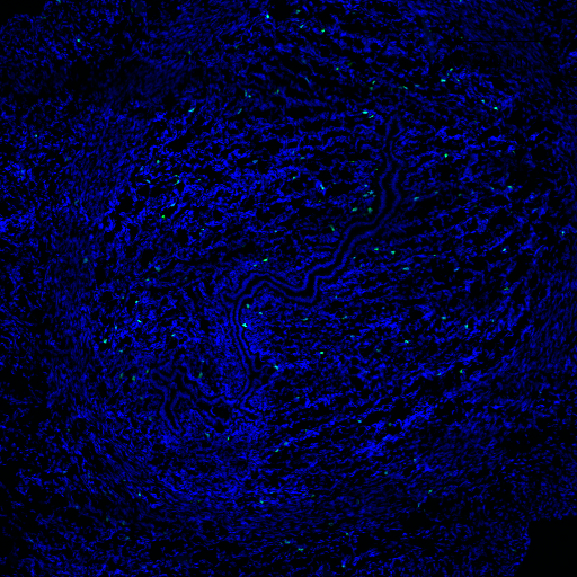

Supplement: Supplementary file 5 — Source Data for Figure 5 [file EMBJ-36-2146-s004.zip › Fig_5B_Source_Figures/5B_T13+ERa.tif]

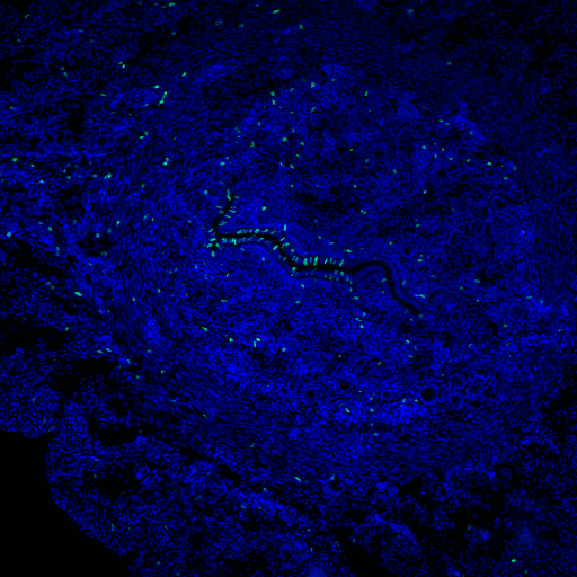

Supplement: Supplementary file 5 — Source Data for Figure 5 [file EMBJ-36-2146-s004.zip › Fig_5B_Source_Figures/5B_T13+Wnt_b-catenin.tif]

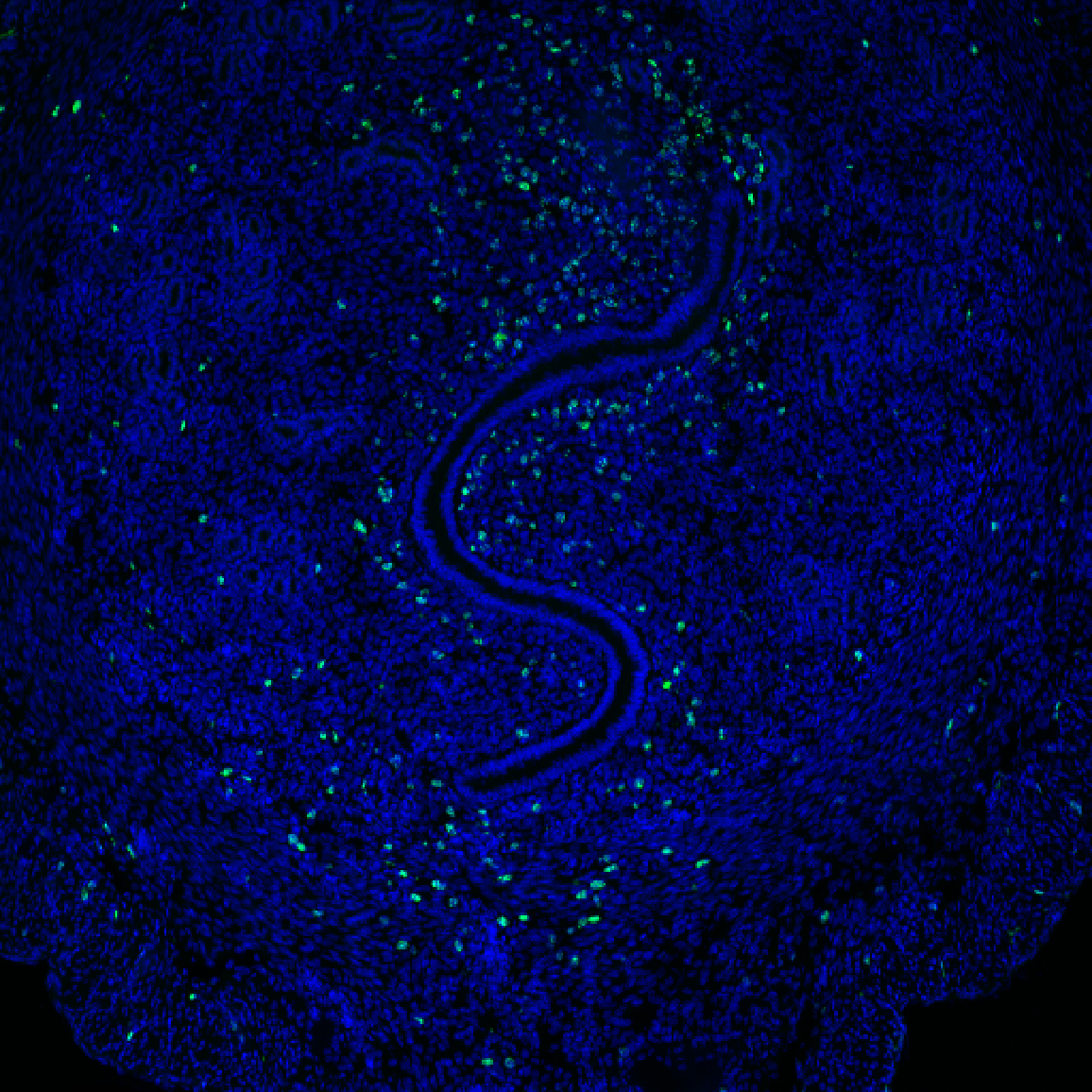

Supplement: Supplementary file 5 — Source Data for Figure 5 [file EMBJ-36-2146-s004.zip › Fig_5B_Source_Figures/5B_vehicle.tiff]
